# Inhaled Cisplatin for NSCLC: Facts and Results

**DOI:** 10.3390/ijms20082005

**Published:** 2019-04-24

**Authors:** Christoforos Kosmidis, Konstantinos Sapalidis, Paul Zarogoulidis, Chrysanthi Sardeli, Charilaos Koulouris, Dimitrios Giannakidis, Efstathios Pavlidis, Athanasios Katsaounis, Nikolaos Michalopoulos, Stylianos Mantalobas, Georgios Koimtzis, Vyron Alexandrou, Theodora Tsiouda, Aikaterini Amaniti, Issak Kesisoglou

**Affiliations:** 1 3rd Department of Surgery, “AHEPA” University Hospital, Aristotle University of Thessaloniki, Medical School, 57001 Thessaloniki, Greece; dr.ckosmidis@gmail.com (C.K.); sapalidiskonstantinos@gmail.com (K.S.); charilaoskoulouris@gmail.com (C.K.); giannakidis.d@gmail.com (D.G.); pavlidis.md@gmail.com (E.P.); athanasios_katsaounis@hotmail.com (A.K.); nmichalopoulos1@outlook.com (N.M.); steliosmantalobas@yahoo.gr (S.M.); drgxkoimtzis@gmail.com (G.K.); vyrwnal@hotmail.com (V.A.); doratsiouda@yahoo.gr (T.T.); amanitik@gmail.com (A.A.); isaackesisoglou@outlook.com (I.K.); 2 Department of Pharmacology & Clinical Pharmacology, School of Medicine, Faculty of Health Sciences, Aristotle University of Thessaloniki, 57001 Thessaloniki, Greece; sardeli@auth.gr

**Keywords:** cisplatin, lung cancer, inhalation, nebulizers, NSCLC

## Abstract

Although we have new diagnostic tools for non-small cell lung cancer, diagnosis is still made in advanced stages of the disease. However, novel treatments are being introduced in the market and new ones are being developed. Targeted therapies and immunotherapy have brought about a bloom in the treatment of non-small cell lung cancer. Still we have to find ways to administer drugs in a more efficient and safe method. In the current review, we will focus on the administration of inhaled cisplatin based on published data.

## 1. Introduction

Lung cancer is still diagnosed at advanced stage in spite of novel diagnostic tools such as the radial endobronchial ultrasound and the convex-probe endobronchial ultrasound (EBUS) [1,2]. Electromagnetic navigation is also a tool for the diagnosis of peripheral small lesions [3]. Lack of early disease symptoms is usually the reason that most patients do not seek early medical attention. Currently we have targeted therapies and immunotherapy for advanced stage disease. Targeted therapy is based on the expression of certain genes such as epidermal growth factor receptor (EGFR), anaplastic lymphoma kinase (ALK), proto-oncogene B-Raf (BRAF), and proto-oncogene tyrosine-protein kinase ROS (ROS-1). Inhibitors are administered in the form of pills for these patients. Regarding immunotherapy this type of therapy is based on the expression of programmed death-ligand 1 (PD-L1). Depending on the expression of PD-L1, there are other drugs that are administered as first line treatment and others as second line treatment [4,5]. Another approach for immunotherapy is the use of monoclonal antibodies (mAbs) to block negative signaling receptors such as CTLA4. This is done by enhancing or prolonging T-cell activation and overcoming tumor-induced immune tolerance. The two drugs ipilimumab and tremelimumab inhibit CTLA4 and at the same time prolong antitumor immune responses, therefore leading to durable anti-tumor effects. Treatment with this novel immunotherapy (mAbs) has demonstrated clinically very important and long-term tumor responses in patients with advanced unresectable cancer disease. In the future, there will be combinations of CTLA-4 blockade therapy that will target several critical regulatory pathways of the immune system and modulate the immune response in the host [6]. However, these combinations might have higher adverse effect rates [7]. Autologous transplantation of immune cells (DCs, tumor specific CTL/NK, modified immune cells such as CAR-T cells) is also being investigated [8]. Currently several methods have been investigated to enhance immunotherapy with the addition of chemotherapy or anti-vascular endothelial growth factor (VEGF) drugs [9,10]. Targeted therapies and immunotherapy have different adverse effects from chemotherapy [11,12,13,14]. In the current review, we will focus on the administration of cisplatin via inhalation with current facts.

## 2. Aerosol Administration

### 2.1. Lung Anatomy

Starting with the mouth and oropharynx afterwards is the trachea. Airways comprise the bronchi, which ultimately reach the alveoli. The main or large bronchial branches have a thick cartilage wall, which when moving to the periphery the thickness progressively diminishes until the branches reach the thin-walled alveoli. The alveoli are easily permeable. There are 23 bronchial divisions before the alveoli are reached [15]. In total, human lungs have a highly vascular surface of (>100 m^2^), where a drug can be absorbed. Mucociliary (beating cilia) are responsible for clearing mucus and drugs included in them. The local absorption depends not only on the thin wall of the alveoli and drug physicochemical properties but also on the local transporters and genes that are expressed locally [16]. In order to have an efficient absorption, dry powder formulations have been developed. There are several ways to produce these formulations: first by precipitation, freeze-drying, or by spray-drying and secondly by micronization via jet milling. These processes involve the use of polymers and lipids or other carrier systems [17,18,19,20,21,22,23,24,25,26,27].

### 2.2. Factors Affecting the Efficacy and Bioavailability of Aerolized Drug Formulations

#### 2.2.1. Inhaled Particle Size

**Figure 1:**
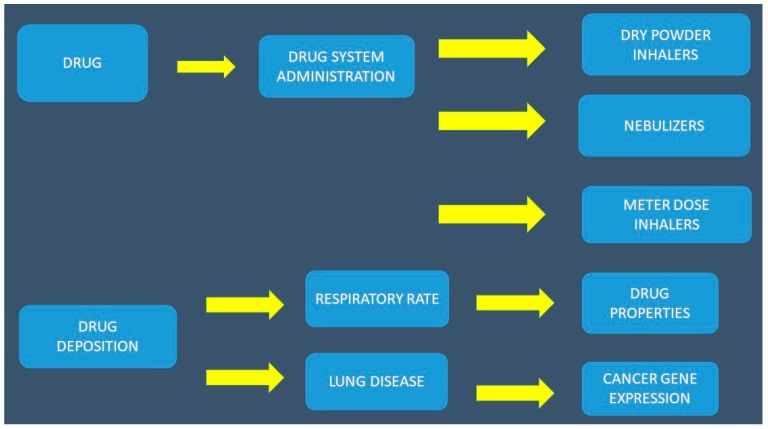
Parameters affecting the optimal drug administration. The dry powder inhalers and meter dose inhalers need specific use with simultaneous coordination of inhalation and device activation for efficient drug deposition. Nebulizers do not need specific device use; they only require deep inhalations and a slow respiratory rate. Drug deposition is affected by local genes in the respiratory tract but also from the tumor genes. Drug properties are also responsible for the local and systematic absorption of an inhaled drug.

The inhaled drug particles should not be larger than 3 μm [17]. Drugs of this size reach the alveoli, are taken up by alveolar epithelial cells, and are carried across and released through the systemic blood stream and interstitial fluid compartment between the epithelial cells [17]. This process is described as transcytosis. There are also drug formulations given via inhalation as bronchodilators and corticosteroids that do not have to be less than 2–3 μm in size, since they act on the large bronchial tubes. Specifically, cisplatin has to be absorbed in the alveoli. The particle or droplet size is measured with master sizer equipment or a cascade impactor and is measured with a mass median aerodynamic diameter (MMAD) (Figure 1).

#### 2.2.2. Airway Geometry and Humidity

Progressive branching and narrowing of the airways encourage the impact of particles. The lung has a high humidity of approximately 99.5%. It is known that all drug particles are hygroscopic, and when they reach the airways they grow or shrink in size based on the humidity of the environment. The increase in particle size above the initial size is very important because larger particles will be deposited in the upper respiratory tract while smaller ones will go deeper [19,20]. The electrophysical properties (Z potential) also play a crucial role in the interaction between the local transporters and local drug absorption mostly in larger airway branches.

#### 2.2.3. Lung Clearance Mechanisms

Drug particles deposited in the conducting airways are primarily removed through mucociliary clearance (beating cilia). The airway epithelial goblet cells and sub-mucosal glands secrete mucus forming a two-layer mucus blanket over the ciliated epithelium: a low-viscosity sol layer covered by a high-viscosity gel layer. Insoluble particles are trapped in a thick mucus layer and are moved towards the pharynx and then lower gastrointestinal tract. In the respiratory tract, the rate of local mucus production and beating cilia movement varies depending on the airway region and underlying respiratory disease. In order to have a normal mucociliary clearance, the airway epithelial cells must be intact, the activity normal of the cilia has to be normal, and the chemical composition of the sol layer must be optimal. These are the basic parameters in order to have an optimal rheology of the mucus within the respiratory tract. In several diseases such as immotile cilia syndrome, bronchiectasis, cystic fibrosis, asthma, and infection by mycoplasma, the mucociliary clearance is impaired [21]. It is known from the physicochemical properties of molecules that lipophilic molecules pass easily through the airway epithelium via passive transport, while on the other hand hydrophilic molecules cross via extracellular pathways and via exocytosis [22]. From the alveoli, the drug particles are absorbed into the systemic circulation and then the lymphatic system. An issue that has to be addressed when creating a new drug or creating an aerosol therapy is alveolar phagocytosis from the local macrophage population. Finally most of the drugs will be absorbed into the pulmonary circulation from the alveoli. Alveolar macrophages are phagocytic cells responsible for the lung defense against all inhaled particles. It has been observed that there are five to seven alveolar macrophages per alveolus in a healthy lung [23]. Macrophages phagocytose insoluble particles that are deposited in the alveolar region. The clearance of the particles is done through the lymphatic system or into the ciliated airways [24]. This process is time-consuming and depends on the respiratory tract status and particle properties [25]. However, regarding proteins, degradation does not occur in total in the alveoli with >95% of proteins, being absorbed intact from the lung periphery [24,26].

#### 2.2.4. Lung Disease

Respiratory diseases, such as cystic fibrosis, bronchiectasis, and emphysema change the architecture of the lung. This occurs due to obstruction from thick mucus and through alterations in bifurcation angles from the destruction of the normal bronchi. In this mode, it modifies the deposition and distribution patterns of aerosols. Respiratory symptoms such as bronchoconstriction and inflammation narrow the airway diameter and alter the particle lung deposition. A decrease in the cross-sectional area of the lung caused by obstruction increases air velocities and turbulence in regions where the airflow is normally laminar. As a result the inspired air is directed to unobstructed airways. Therefore, only a very small quantity of the drug is deposited on the surface of the obstructed areas. Usually these areas need to be reached in order to achieve the optimal therapeutic effect of the drug [21].

#### 2.2.5. Bronchial Circulation

The lungs receive the entire cardiac output and represent the most richly perfused organ in the body. It is known that only the alveolar region is supplied by the pulmonary circulation. Blood flow to the larger airways such as the trachea and bronchi is via the systemic circulation. Based on the human anatomy, these airways receive approximately 1% of the total cardiac output [27]. The endobronchial circulation is re-circulated to the peripheral airways and lung parenchyma via the bronchial veins and right atrium. It has been observed that the bronchial blood flow increases in respiratory diseases, from 1 to 30% of the total cardiac output. An inhaled drug can be absorbed into the circulation from the tracheobronchial regions and redistributed downstream to the periphery and into otherwise poorly accessible areas of the lung. These findings aid the drug’s efficacy and should be kept in mind when creating an aerosol therapy [27]. Moreover, further drug absorption occurs via the lymphatic pathway [28,29].

#### 2.2.6. Duration of Inhalation, Drugs, Breathing Frequency (F) and Tidal Volume (TV), Local Cells, the Local Environment, and Aerosol Production Systems

**Figure 2:**
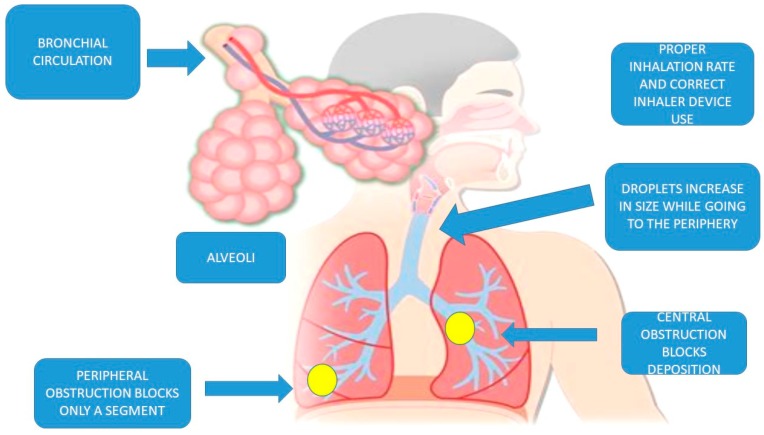
Schematic of parameters affecting the optimal administration and where in the airways. The correct use of an inhaler along with the correct inhalation force is essential for the best drug deposition. A central obstruction blocks the deposition of the aerosol drug in a lung or lobe, while a peripheral obstruction blocks a lobe or only a small segment of the lobe. The bronchial circulation in the alveoli is responsible for the systematic absorption of the inhaled drug.

These three factors affect the absorption of a drug formulation in every breath. There are methods to alter TV and F, which are described below [30,31]. The administration of aerosol bronchodilators and corticosteroids are used by several patients with respiratory diseases, such as chronic obstructive pulmonary disease (COPD), asthma, cystic fibrosis, bronchiectasis, and lung infection [32,33,34]. Bronchodilators are known to dilate the airways that are narrowed due to the underlying respiratory disease, while the corticosteroids reduce and block the inflammation locally. These drugs therefore assist in a more efficient drug circulation and deposition. There are several aerosol production systems such as ultrasound nebulizers and jet-nebulizers. Moreover, there are aerosol delivery systems that rely on the respiratory performance of the patients such as metered dose inhalers (MDIs) and dry powder inhalers (DPIs) [35]. Every aerosol delivery system has its advantages and disadvantages. Depending on the patients’ underlying disease and respiratory performance, the medical doctor has to decide which delivery system and administration method is the most appropriate. The production systems of ultrasound nebulizers and jet-nebulizers do not require the coordination between patient and administration as in the case of MDIs. Furthermore, the misuse of DPIs might lead to blowing the drug into the device. Moreover, local cell structures and cells such as enzymes, mucus, local transporters/genes, beating cilia, and macrophages play a crucial role in the absorption of inhaled drugs [36]. It is known that the humidity of the respiratory system is almost 95%, and it is responsible for expanding droplets and particles [37]. It has been observed that a drug can expand up to 50% of the initial size. The optimal size for deep penetration and deposition into the lung parenchyma is between 3 and 5 μm mass median aerodynamic diameter (MMAD). The salts of a solution are also responsible for the expansion of the droplet size. The higher the salt concentration, the higher the droplet expansion [38]. It has been observed for the jet nebulizers the main factors affecting the droplet size are (a) the flow rate, (b) the residual cup design, (c) the drug (the viscosity and electrostatic charge), (d) the mouthpiece design, and (e) the residual cup loading [37,39,40,41,42,43,44]. It has been observed that the time of nebulization depends on the volume in the residual cup; specifically, the higher the volume, the shorter the nebulization time [45]. Therefore, one could refill the residual cup when the volume of the solution reaches half of the initial fill; however, this method can only be applied once [46]. The factors affecting the ultrasound nebulizers are (a) the addition of buffer, (b) the time of nebulization, (c) the temperature of the piezoelectric crystal, and (d) the drug (the salts and viscosity) [47] [45,46,48,49,50]. The inlet of the aerosol production systems has also influenced droplet size production [51]. Moreover, the addition of 5–7% CO_2_ gas during an aerosol administration increases the tidal volume by 180% and reduces the respiratory frequency. The result is deep and slow breaths (Figure 2).

## 3. Cisplatin Studies

One of the first studies to be performed as Phase I with liposomal cisplatin was the study by Wittgen B. et al. [52]. Sixteen non-small cell lung cancer (NSCLC) patients and 1 with small cell lung cancer (SCLC) were included. Respiratory adverse effects were monitored with pulmonary function tests (PFTs) and imaging techniques, specifically CTs of the thorax. Moreover, systemic adverse effects were monitored. A dose escalation was performed during the treatment. This study was one of the first to demonstrate a proof of concept. The respiratory adverse effects recorded were due to the physicochemical properties of the liposomal cisplatin, and grade 2 was not exceeded in any case. Non-specific bronchitis, nausea, and fatigue were observed after the jet-nebulizer cisplatin administration. The administration of the aerosol in one patient was performed through his tracheostoma. Liposomes containing anticancer drugs have been extensively investigated and are considered a very efficient carrier; however, an efficient delivery system should accompany the drug formulation [53]. In a study by Tseng C.L. et al. [54], gelatin nanoparticles loaded with cisplatin were developed and decorated with epidermal growth factor (EGF) tumor-specific ligand. Their in vitro and in vivo targeting ability and anticancer effect were confirmed. Moreover, the aerosol delivery of the nanodrug carrier was demonstrated both in vitro and in vivo in mice. Additional equipment could be used to further enhance the aerosol drug delivery. The main issue was weight loss for the animals.

The group of Lee H.-Y. et al. [55] developed cisplatin loaded albumin mesospheres for lung cancer treatment. Albumin mesospheres have excellent physicochemical properties and are an efficient matrix for “metal” drugs such as the platinum analogs. In their study, the specific compound was produced for intratumoral application (liquid) or was administered as aerosol (dry powder). Its efficacy was evaluated in vitro. The combination of two chemotherapy drugs in one solution, paclitaxel and cisplatin, was investigated by El-Gendy N. et al. [56]; specifically the amino acid, l-leucine, was used as a colloid destabilizer to drive the assembly of paclitaxel nanoparticles. Afterwards, a combination chemotherapy aerosol was formed by assembling the paclitaxel nanoparticles in the presence of cisplatin in solution. The study also evaluated the performance of the drug in vitro, and the results indicated that the nanoparticle agglomerate dry powders exhibited aerosol characteristics and size distributions appropriate for pulmonary drug delivery. Hyaluronan–cisplatin conjugate nanoparticles were created by Ishiguro S. et al. [57] and tested via intratracheal instillation in mice. We include this study in our manuscript since the same solution could be applied as aerosol if it were used in a nebulizer, or with a dry freezer if it were converted to dry powder. Regarding the effectiveness of the compound cancer stem cells and cisplatin resistant cells marker, CD44 expression decreased in the tumor nodules of the HA-Pt but not in those of cisplatin treated groups. In the study by Selting K. et al. [58], cisplatin was administered with the help of an intracorporal catheter to the airways of dogs, cough, pneumonitis, and fibrotic lesions were observed; however, these findings were attributed to the large concentrations of the drug that was locally gathered in segments. By this method, which was instillation of the drug in the lungs, the distribution was not proper throughout the airways and lung parenchyma. Specifically, large quantities of the drug were deposited in different areas instead of the whole airways, as is the case with an aerosol.

Nanoparticles of cisplatin in three forms were used to enhance radiotherapy application, specifically cisplatin nanoparticles (CNPs), carboplatin nanoparticles (CBNPs), and gold nanoparticles (GNPs) [59]. The results indicated that major dose enhancement to lung tumors can be achieved by using GNPs, CNPs, and CBNPs administered via the inhalational route (IR), when compared to the intravenous administration of these agents during external beam radiotherapy (EBRT). In this study, however, we do not have reports of pulmonary function test measurements or of observation of the pulmonary parenchyma with imaging techniques. Therefore, further evaluation is needed. In a study by Zarogoulidis P. et al. [60], sixty NSCLC patients were included in three different groups. Evaluation was performed with PFTs and CT imaging. Preparation with inhaled corticosteroids was done to the patients of the inhalation groups. There were no respiratory adverse effects more than grade 2, which were all observed right after the inhalation in the form of bronchitis, nausea, and fatigue. All the adverse effects were monitored up to six months, so we do not have data after this time. Based on the included cisplatin studies, we can comment that there are very few studies performed on patients; however, the pulmonary and imaging findings do not indicate severe adverse effects at least up to six months. Moreover, the pharmacokinetics of the inhaled cisplatin was observed with blood samples at different time limits. In another study by Zarogoulidis P. et al. [61], stage II NSCLC patients received inhaled cisplatin right before surgery. The particles of cisplatin were observed within the lymphnodes with the help of CytoViva^®^ (Auburn, AL, USA). This study is proof of concept that an inhaled drug reaches the lymphatic circulation. Thus, an inhalational administration becomes systematic after several minutes. Chitosan-based dry powder was developed by Singh D. et al. [62] and was tested in vitro. Chou A. J. et al. [63] performed a study with osteosarcoma patients with inhaled lipid cisplatin. We refer to this study because it is the only study with a patient presenting with grade 3 adverse effects after inhalation. Inhaled cisplatin was compared to inhaled doxorubicin with doxorubicin being more effective. However, more adverse effects were observed in the inhaled doxorubicin group [16,64]. Taratula O. et al. targeted both local airway genes/transporters and the drug administration in a single molecule [65]. Local delivery of mesoporous silica nanoparticles (MSNs) by inhalation led to the accumulation of nanoparticles in mouse lungs locally and prevented the absorption of MSNs into the systemic circulation or lymphatic circulation. The group of Levet V. et al. [66] published one of the last studies with dry-powder cisplatin with a sustained release activity. The compound that was developed is a breakthrough for the drug industry due to its physicochemical properties and the mass median aerodynamic diameter (MMAD) of the powder size. It remains to be tested in vivo. There have been other studies with other chemotherapeutic agents such as doxorubicin, gemcitabine, paclitaxel, 5-fluorouracil, and 9-nitro-camptothecin [16]. Doxorubicin, gemcitabine, and paclitaxel had the most severe pulmonary and imaging side effects, but these effects can be also attributed to the drugs’ physicochemical properties (Table 1).

## 4. Current and Future Administration Strategies

Current administration of cisplatin has been performed with jet nebulizers and instillation through catheters. In other studies with other chemotherapeutic drugs, the administration was performed with some modifications such as the addition of CO_2_. By doing this, the respiratory frequency decreased, and the tidal volume increased, causing the subject to inhale more slowly, taking in higher volumes of air [16]. Cisplatin aerosol equipment has been also constructed as pressurized intraperitoneal aerosol chemotherapy (PIPAC) and administered endoperitoneally in metastatic disease from several cancers. We refer to this study because it contains an apparatus that can deliver aerosol cisplatin [67]. In the study by Anderson K. et al. [68], a novel nonhydrolyzable ether-linked acetic acid analog of vitamin E, 2,5,7,8-tetramethyl-2*R*-(4*R*,8*R*,12-trimethyltridecyl)-chroman-6-yloxyacetic acid (α-TEA), was combined with cisplatin and reduced the tumor burden of A2780/cp70 (cp70) cisplatin-resistant human ovarian cancer cells xenografted into immune compromised nude mice. Moreover, creating molecules that can target, at the same time, local transporters and genes in the airway is possible and should be pursued [65]. Another method of enhancing and producing a sustained release effect is by creating “stealth” molecules that can bypass the cleaning mechanism either locally in the airways or in the blood stream [16]. Apart from the methods of administration, we should keep in mind that cancer has its own mechanisms and “environment,” and that is why we observe therapy resistance and cancer gene expression modification after several cycles of therapy administration with different drugs [69]. Finally, apart from an efficient drug and drug administration, we should consider the combination of drug treatments such as immunotherapy and anti-angiogenetic agents [9]. Pretreatment with radiotherapy or chemotherapy could also enhance several therapies such as in the case of immunotherapy [70,71]. Since inhaled chemotherapeutic compounds have presented toxicity, although most of them were in mild forms and observed with PFTs and CT imaging, prophylaxis with inhaled steroids should be considered when designing such a study, as has been previously proposed [72,73,74]. Finally, we should consider creating a device that can actually measure the forced expiratory volume in 1 s (FEV_1_) with software and then provide the drug in a mode specific to each patient. Moreover, we should consider upgrading the current devices with novel mouthpiece designs that actually enhance the properties of the droplets or drug dry powder [40].

## 5. Conclusions

There is currently a variety of inhalational devices available on the market, and each one has its advantages and disadvantages. To date administration of an inhaled chemotherapy formulation has been performed to humans through nebulization systems (jet nebulizers). The aerosol compound time release can be enhanced by either adding a carrier that provides sustained release, or by adding 5–7% CO_2_ to the inhalable aerosol [16]. Most of the aerosol cisplatin studies previously published provide conclusions with safety and feasibility of this treatment modality. However, clinical trials with patients of early stages (stage II and IIIA) are needed to present long-term data regarding adverse effects to the lung parenchyma. In addition, more double-agent trials with aerosol chemotherapies are needed to present indisputable data regarding the safety and effectiveness of this treatment modality in comparison with intravenous administration. Moreover, we have to consider that this treatment modality could be used as a neoadjuvant/adjuvant, since tumor size is a limitation for patients to be candidates. In the future, we could administer inhalational chemotherapy in house with proper education of the patients. Administration of inhaled bronchodilators, corticosteroids, and N-acetylcysteine could prevent and protect the lung parenchyma from adverse effects as indicated in previous studies [74].

The major advantage of local treatment in the form of aerosol is the fast and increased local concentration not only to the target site but also in other parts of the lung parenchyma where carcinogenesis might be initiated. The increased local concentration then becomes systematic since the drug is absorbed locally through the alveoli and again cancer cells can be destroyed in the blood circulation. Unfortunately, some drugs in their current form are toxic for the lung parenchyma and cannot be administered until they are modified. Moreover, instillation cannot be considered the correct form of administration because high local concentrations (different concentrations for every agent) can induce local toxic effects, while by aerosol administration these would not be observed with the same agent and concentration. The concept of a treatment modality for cancer patients free of systemic side effects is enticing and should be pursued.

## Figures and Tables

**Table 1:** Inhalation studies.

Preclinical	- The use of biotinylated-EGF-modified gelatin nanoparticle carrier to enhance cisplatin accumulation in cancerous lungs via inhalation. - Cisplatin loaded albumin mesospheres for lung cancer treatment. - Combination chemotherapeutic dry powder aerosols via controlled nanoparticle agglomeration. - Intratracheal administration of hyaluronan–cisplatin conjugate nanoparticles significantly attenuates lung cancer growth in mice. - Targeted combined aerosol chemotherapy in dogs and radiologic toxicity grading. - Development of chitosan-based dry powder inhalation system of cisplatin for lung cancer. - Innovative strategy for treatment of lung cancer: targeted nanotechnology-based inhalation co-delivery of anticancer drugs and siRNA. - Development of controlled-release cisplatin dry powders for inhalation against lung cancers.
Clinical	- Phase I study of aerosolized SLIT cisplatin in the treatment of patients with carcinoma of the lung. - Potential for enhancing external beam radiotherapy for lung cancer using high-Z nanoparticles administered via inhalation. - Feasibility and effectiveness of inhaled carboplatin in NSCLC patients. - Inhaled cisplatin deposition and distribution in lymph nodes in stage II lung cancer patients. - Inhaled lipid cisplatin (ILC) in the treatment of patients with relapsed/progressive osteosarcoma metastatic to the lung. - Comparison of quick recovery outcome of inhalable doxorubicin and cisplatin in lung cancer patients: a randomized, double-blind, single-center trial.

## References

[1] Zaric B., Stojsic V., Carapic V., Kovacevic T., Stojanovic G., Panjkovic M., Kioumis I., Darwiche K., Zarogoulidis K., Stratakos G. (2016). Radial Endobronchial Ultrasound (EBUS) Guided Suction Catheter-Biopsy in Histological Diagnosis of Peripheral Pulmonary Lesions. J. Cancer.

[2] Oezkan F., Khan A., Zarogoulidis P., Hohenforst-Schmidt W., Theegarten D., Yasufuku K., Nakajima T., Freitag L., Darwiche K. (2014). Efficient utilization of EBUS-TBNA samples for both diagnosis and molecular analyses. OncoTargets Ther..

[3] Zaric B., Kovacevic T., Stojsic V., Milovancev A. (2016). New technologies in diagnostic bronchoscopy—an age of meta-analyses. Expert Rev. Med. Devices.

[4] Zarogoulidis P., Papadopoulos V., Maragouli E., Papatsibas G., Sardeli C., Man Y.G., Bai C., Huang H. (2018). Nivolumab as first-line treatment in non-small cell lung cancer patients-key factors: tumor mutation burden and PD-L1 >/=50. Transl. Lung Cancer Res..

[5] Rolfo C., Caglevic C., Santarpia M., Araujo A., Giovannetti E., Gallardo C.D., Pauwels P., Mahave M. (2017). Immunotherapy in NSCLC: A Promising and Revolutionary Weapon. Adv. Exp. Med. Biol..

[6] Tarhini A.A., Iqbal F. (2010). CTLA-4 blockade: therapeutic potential in cancer treatments. OncoTargets Ther..

[7] Hassel J.C., Heinzerling L., Aberle J., Bahr O., Eigentler T.K., Grimm M.O., Grunwald V., Leipe J., Reinmuth N., Tietze J.K. (2017). Combined immune checkpoint blockade (anti-PD-1/anti-CTLA-4): Evaluation and management of adverse drug reactions. Cancer Treat. Rev..

[8] Hirsch F.R., Scagliotti G.V., Mulshine J.L., Kwon R., Curran W.J., Wu Y.L., Paz-Ares L. (2017). Lung cancer: current therapies and new targeted treatments. Lancet.

[9] Manegold C., Dingemans A.C., Gray J.E., Nakagawa K., Nicolson M., Peters S., Reck M., Wu Y.L., Brustugun O.T., Crino L. (2017). The Potential of Combined Immunotherapy and Antiangiogenesis for the Synergistic Treatment of Advanced NSCLC. J. Thoracic Oncol..

[10] Insinga R.P., Vanness D.J., Feliciano J.L., Vandormael K., Traore S., Ejzykowicz F., Burke T. (2019). Cost-effectiveness of pembrolizumab in combination with chemotherapy versus chemotherapy and pembrolizumab monotherapy in the first-line treatment of squamous non-small-cell lung cancer in the US. Curr. Med. Res. Opin..

[11] Zarogoulidis P., Chinelis P., Athanasiadou A., Porpodis K., Kallianos A., Rapti A., Trakada G., Velentza L., Huang H., Tsiouda T. (2017). “Liquid elbows” due to afatinib administration. Respir. Med. Case Rep..

[12] Sapalidis K., Kosmidis C., Michalopoulos N., Koulouris C., Mantalobas S., Giannakidis D., Munteanu A., Surlin V., Laskou S., Zarogoulidis P. (2018). Psoriatic arthritis due to nivolumab administration a case report and review of the literature. Respir. Med. Case Rep..

[13] Zarogoulidis P., Huang H., Tsiouda T., Sardeli C., Trakada G., Veletza L., Kallianos A., Kosmidis C., Rapti A., Papaemmanouil L. (2017). Immunotherapy “Shock” with vitiligo due to nivolumab administration as third line therapy in lung adenocarcinoma. Respir. Med. Case Rep..

[14] Livshits Z., Rao R.B., Smith S.W. (2014). An approach to chemotherapy-associated toxicity. Emerg. Med. Clin. North Am..

[15] Ray A., Mandal A., Mitra A.K. (2015). Recent Patents in Pulmonary Delivery of Macromolecules. Recent Pat. Drug Deliv. Formul..

[16] Zarogoulidis P., Chatzaki E., Porpodis K., Domvri K., Hohenforst-Schmidt W., Goldberg E.P., Karamanos N., Zarogoulidis K. (2012). Inhaled chemotherapy in lung cancer: future concept of nanomedicine. Int. J. Nanomed..

[17] Labiris N.R., Dolovich M.B. (2003). Pulmonary drug delivery. Part I: physiological factors affecting therapeutic effectiveness of aerosolized medications. Br. J. Clin. Pharm..

[18] Labiris N.R., Dolovich M.B. (2003). Pulmonary drug delivery. Part II: the role of inhalant delivery devices and drug formulations in therapeutic effectiveness of aerosolized medications. Br. J. Clin. Pharm..

[19] Phipps P.R., Gonda I., Anderson S.D., Bailey D., Bautovich G. (1994). Regional deposition of saline aerosols of different tonicities in normal and asthmatic subjects. Eur. Respir. J..

[20] Swift D.L. (1980). Aerosols and humidity therapy. Generation and respiratory deposition of therapeutic aerosols. Am. Rev. Respir. Dis..

[21] Houtmeyers E., Gosselink R., Gayan-Ramirez G., Decramer M. (1999). Regulation of mucociliary clearance in health and disease. Eur. Respir. J..

[22] Summers Q.A. (1991). Inhaled drugs and the lung. Clin. Exp. Allergy.

[23] Wirkes A., Jung K., Ochs M., Muhlfeld C. (2010). Allometry of the mammalian intracellular pulmonary surfactant system. J. Appl. Physiol..

[24] Folkesson H.G., Matthay M.A., Westrom B.R., Kim K.J., Karlsson B.W., Hastings R.H. (1996). Alveolar epithelial clearance of protein. J. Appl. Physiol..

[25] Martonen T.B. (1993). Mathematical model for the selective deposition of inhaled pharmaceuticals. J. Pharm. Sci..

[26] Hastings R.H., Grady M., Sakuma T., Matthay M.A. (1992). Clearance of different-sized proteins from the alveolar space in humans and rabbits. J. Appl. Physiol..

[27] Deffebach M.E., Charan N.B., Lakshminarayan S., Butler J. (1987). The bronchial circulation. Small, but a vital attribute of the lung. Am. Rev. Respir. Dis..

[28] Shinohara H. (1997). Distribution of lymphatic stomata on the pleural surface of the thoracic cavity and the surface topography of the pleural mesothelium in the golden hamster. Anat. Rec..

[29] Lai-Fook S.J. (1993). Mechanical factors in lung liquid distribution. An. Rev. Physiol..

[30] Koshkina N.V., Knight V., Gilbert B.E., Golunski E., Roberts L., Waldrep J.C. (2001). Improved respiratory delivery of the anticancer drugs, camptothecin and paclitaxel, with 5% CO_2_-enriched air: Pharmacokinetic studies. Cancer Chemother. Pharm..

[31] Davis J.N., Stagg D. (1975). Interrelationships of the volume and time components of individual breaths in resting man. J. Physiol..

[32] Dekhuijzen P.N., Vincken W., Virchow J.C., Roche N., Agusti A., Lavorini F., van Aalderen W.M., Price D. (2013). Prescription of inhalers in asthma and COPD: Towards a rational, rapid and effective approach. Respir. Med..

[33] Mogayzel P.J., Naureckas E.T., Robinson K.A., Mueller G., Hadjiliadis D., Hoag J.B., Lubsch L., Hazle L., Sabadosa K., Marshall B. (2013). Cystic fibrosis pulmonary guidelines. Chronic medications for maintenance of lung health. Am. J. Respir. Crit. Care Med..

[34] Chang A.B., Marsh R.L., Smith-Vaughan H.C., Hoffman L.R. (2012). Emerging drugs for bronchiectasis. Expert Opin. Emerg. Drugs.

[35] Laube B.L., Janssens H.M., de Jongh F.H., Devadason S.G., Dhand R., Diot P., Everard M.L., Horvath I., Navalesi P., Voshaar T. (2011). What the pulmonary specialist should know about the new inhalation therapies. Eur. Respir. J..

[36] Zarogoulidis P., Darwiche K., Spyratos D., Secen N., Hohenforst-Schmidt W., Katsikogiannis N., Huang H., Gschwendtner A., Zarogoulidis K. (2014). Defense Mechanisms of the Respiratory System and Aerosol Production Systems. Med. Chem..

[37] Clay M.M., Pavia D., Newman S.P., Clarke S.W. (1983). Factors influencing the size distribution of aerosols from jet nebulisers. Thorax.

[38] Bier M., de Graaf J., Zwanikken J., van Roij R. (2009). Curvature dependence of the electrolytic liquid-liquid interfacial tension. J. Chem. Physiol..

[39] Zarogoulidis P., Petridis D., Ritzoulis C., Darwiche K., Spyratos D., Huang H., Goldberg E.P., Yarmus L., Li Q., Freitag L. (2013). Establishing the optimal nebulization system for paclitaxel, docetaxel, cisplatin, carboplatin and gemcitabine: back to drawing the residual cup. Int. J. Pharm..

[40] Zarogoulidis P., Petridis D., Ritzoulis C., Darwiche K., Kioumis I., Porpodis K., Spyratos D., Hohenforst-Schmidt W., Yarmus L., Huang H. (2013). Internal mouthpiece designs as a future perspective for enhanced aerosol deposition. Comparative results for aerosol chemotherapy and aerosol antibiotics. Int. J. Pharm..

[41] Mercer T.T., Goddard R.F., Flores R.L. (1969). Effect of auxiliary air flow on the output characteristics of compressed-air nebulizers. Ann. Allergy.

[42] Kendrick A.H., Smith E.C., Wilson R.S. (1997). Selecting and using nebuliser equipment. Thorax.

[43] Newman S.P., Pellow P.G., Clay M.M., Clarke S.W. (1985). Evaluation of jet nebulisers for use with gentamicin solution. Thorax.

[44] Sterk P.J., Plomp A., van de Vate J.F., Quanjer P.H. (1984). Physical properties of aerosols produced by several jet- and ultrasonic nebulizers. Bull Eur. Physiopathol. Respir..

[45] Ferron G.A., Kerrebijn K.F., Weber J. (1976). Properties of aerosols produced with three nebulizers. Am. Rev. Respir. Dis..

[46] Steckel H., Eskandar F. (2003). Factors affecting aerosol performance during nebulization with jet and ultrasonic nebulizers. Eur. J. Pharm. Sci..

[47] Lourenco R.V., Cotromanes E. (1982). Clinical aerosols II. Therapeutic aerosols. Arch Intern. Med..

[48] Niven R.W., Ip A.Y., Mittelman S., Prestrelski S.J., Arakawa T. (1995). Some factors associated with the ultrasonic nebulization of proteins. Pharm. Res..

[49] Dennis J.H., Stenton S.C., Beach J.R., Avery A.J., Walters E.H., Hendrick D.J. (1990). Jet and ultrasonic nebuliser output: use of a new method for direct measurement of aerosol output. Thorax.

[50] O’Callaghan C., Barry P.W. (1997). The science of nebulised drug delivery. Thorax.

[51] Darwiche K., Zarogoulidis P., Baehner K., Welter S., Tetzner R., Wohlschlaeger J., Theegarten D., Nakajima T., Freitag L. (2013). Assessment of SHOX2 methylation in EBUS-TBNA specimen improves accuracy in lung cancer staging. Ann. Oncol..

[52] Wittgen B.P., Kunst P.W., van der Born K., van Wijk A.W., Perkins W., Pilkiewicz F.G., Perez-Soler R., Nicholson S., Peters G.J., Postmus P.E. (2007). Phase I study of aerosolized SLIT cisplatin in the treatment of patients with carcinoma of the lung. Clin. Cancer Res..

[53] Rudokas M., Najlah M., Alhnan M.A., Elhissi A. (2016). Liposome Delivery Systems for Inhalation: A Critical Review Highlighting Formulation Issues and Anticancer Applications. Med. Princ. Pract..

[54] Tseng C.L., Su W.Y., Yen K.C., Yang K.C., Lin F.H. (2009). The use of biotinylated-EGF-modified gelatin nanoparticle carrier to enhance cisplatin accumulation in cancerous lungs via inhalation. Biomaterials.

[55] Lee H.Y., Mohammed K.A., Goldberg E.P., Kaye F., Nasreen N. (2015). Cisplatin loaded albumin mesospheres for lung cancer treatment. Am. J. Cancer Res..

[56] El-Gendy N., Berkland C. (2009). Combination chemotherapeutic dry powder aerosols via controlled nanoparticle agglomeration. Pharm. Res..

[57] Ishiguro S., Cai S., Uppalapati D., Turner K., Zhang T., Forrest W.C., Forrest M.L., Tamura M. (2016). Intratracheal Administration of Hyaluronan-Cisplatin Conjugate Nanoparticles Significantly Attenuates Lung Cancer Growth in Mice. Pharm. Res..

[58] Selting K., Essman S., Reinero C., Branson K.R., Henry C.J., Owen N., Guntur V.P., Waldrep J.C., Kim D.Y., Dhand R. (2011). Targeted combined aerosol chemotherapy in dogs and radiologic toxicity grading. J. Aerosol Med. Pulm. Drug Deliv..

[59] Hao Y., Altundal Y., Moreau M., Sajo E., Kumar R., Ngwa W. (2015). Potential for enhancing external beam radiotherapy for lung cancer using high-Z nanoparticles administered via inhalation. Physiol. Med. Biol..

[60] Zarogoulidis P., Eleftheriadou E., Sapardanis I., Zarogoulidou V., Lithoxopoulou H., Kontakiotis T., Karamanos N., Zachariadis G., Mabroudi M., Zisimopoulos A. (2012). Feasibility and effectiveness of inhaled carboplatin in NSCLC patients. Invest. New Drugs.

[61] Zarogoulidis P., Darwiche K., Krauss L., Huang H., Zachariadis G.A., Katsavou A., Hohenforst-Schmidt W., Papaiwannou A., Vogl T.J., Freitag L. (2013). Inhaled cisplatin deposition and distribution in lymph nodes in stage II lung cancer patients. Fut. Oncol..

[62] Singh D.J., Lohade A.A., Parmar J.J., Hegde D.D., Soni P., Samad A., Menon M.D. (2012). Development of Chitosan-based Dry Powder Inhalation System of Cisplatin for Lung Cancer. Indian J. Pharm. Sci..

[63] Chou A.J., Gupta R., Bell M.D., Riewe K.O., Meyers P.A., Gorlick R. (2013). Inhaled lipid cisplatin (ILC) in the treatment of patients with relapsed/progressive osteosarcoma metastatic to the lung. Pediatr. Blood Cancer.

[64] Li Z., Song M., He Z., Zong L., Jiang B., Zhang T., Hu Z. (2018). Comparison of quick recovery outcome of inhalable doxorubicin and cisplatin in lung cancer patients: a randomized, double-blind, single-center trial. Drug Deliv. Transl. Res..

[65] Taratula O., Garbuzenko O.B., Chen A.M., Minko T. (2011). Innovative strategy for treatment of lung cancer: targeted nanotechnology-based inhalation co-delivery of anticancer drugs and siRNA. J. Drug Target..

[66] Levet V., Rosiere R., Merlos R., Fusaro L., Berger G., Amighi K., Wauthoz N. (2016). Development of controlled-release cisplatin dry powders for inhalation against lung cancers. Int. J. Pharm..

[67] Nowacki M., Alyami M., Villeneuve L., Mercier F., Hubner M., Willaert W., Ceelen W., Reymond M., Pezet D., Arvieux C. (2018). Multicenter comprehensive methodological and technical analysis of 832 pressurized intraperitoneal aerosol chemotherapy (PIPAC) interventions performed in 349 patients for peritoneal carcinomatosis treatment: An international survey study. Eur. J. Surg. Oncol..

[68] Anderson K., Lawson K.A., Simmons-Menchaca M., Sun L., Sanders B.G., Kline K. (2004). Alpha-TEA plus cisplatin reduces human cisplatin-resistant ovarian cancer cell tumor burden and metastasis. Exp. Biol. Med..

[69] Rezniczek G.A., Jungst F., Jutte H., Tannapfel A., Hilal Z., Hefler L.A., Reymond M.A., Tempfer C.B. (2016). Dynamic changes of tumor gene expression during repeated pressurized intraperitoneal aerosol chemotherapy (PIPAC) in women with peritoneal cancer. BMC Cancer.

[70] Masucci G.V., Cesano A., Hawtin R., Janetzki S., Zhang J., Kirsch I., Dobbin K.K., Alvarez J., Robbins P.B., Selvan S.R. (2016). Validation of biomarkers to predict response to immunotherapy in cancer: Volume I - pre-analytical and analytical validation. J. Immunother. Cancer.

[71] Turgeon G.A., Weickhardt A., Azad A.A., Solomon B., Siva S. (2019). Radiotherapy and immunotherapy: a synergistic effect in cancer care. Med. J. Austr..

[72] McGaughey D.S., Nikcevich D.A., Long G.D., Vredenburgh J.J., Rizzieri D., Smith C.A., Broadwater G., Loftis J.S., McDonald C., Morris A.K. (2001). Inhaled steroids as prophylaxis for delayed pulmonary toxicity syndrome in breast cancer patients undergoing high-dose chemotherapy and autologous stem cell transplantation. Biol. Blood Marrow Transpl..

[73] Darwiche K., Zarogoulidis P., Karamanos N.K., Domvri K., Chatzaki E., Constantinidis T.C., Kakolyris S., Zarogoulidis K. (2013). Efficacy versus safety concerns for aerosol chemotherapy in non-small-cell lung cancer: a future dilemma for micro-oncology. Fut. Oncol..

[74] Zarogoulidis P., Giraleli C., Karamanos N.K. (2012). Inhaled chemotherapy in lung cancer: safety concerns of nanocomplexes delivered. Ther. Deliv..

